# Rate of revision and wear penetration in different polyethylene liner compositions in total hip arthroplasty: a Bayesian network meta-analysis

**DOI:** 10.1038/s41598-024-71326-1

**Published:** 2024-09-10

**Authors:** Filippo Migliorini, Marcel Betsch, Nicola Maffulli, Luise Schäfer, Frank Hildebrand, Joshua Kubach, Mario Pasurka

**Affiliations:** 1 Department of Orthopaedic, Trauma, and Reconstructive Surgery, RWTH University Hospital, Pauwelsstraße 30, 52074 Aachen, Germany; 2 Department of Orthopedics and Trauma Surgery, Academic Hospital of Bolzano (SABES-ASDAA), 39100 Bolzano, Italy; 3 Department of Life Sciences, Health, and Health Professions, Link Campus University, 00165 Rome, Italy; 4 Department of Orthopaedics and Trauma Surgery, University Hospital Erlangen, 91054 Erlangen, Germany; 5 Department of Medicine and Psychology, University of Rome “La Sapienza”, Rome, Italy; 6 School of Pharmacy and Bioengineering, Faculty of Medicine, Keele University, Stoke on Trent, ST4 7QB UK; 7 Centre for Sports and Exercise Medicine, Barts and the London School of Medicine and Dentistry, Mile End Hospital, Queen Mary University of London, London, E1 4DG UK

**Keywords:** Hip, Arthroplasty, THA, Liner, Polyethylene, Wear, Revision, Medical research, Outcomes research

## Abstract

The present Bayesian network meta-analysis compared different types of polyethylene liners in total hip arthroplasty (THA) in terms of wear penetration (mm/year) and rate of revision. The type of liners compared were the crosslinked ultra-high molecular weight polyethylene (CPE/UHMWPE), Vitamin E infused highly cross-linked polyethylene (HXLPE-VEPE), modified cross-linked polyethylene (MXLPE), highly cross-linked polyethylene (HXLPE), Cross-linked polyethylene (XLPE). This study was conducted according to the PRISMA extension statement for reporting systematic reviews incorporating network meta-analyses of healthcare interventions. In June 2024, PubMed, Scopus, Embase, Google Scholar, and Cochrane databases were accessed. A time constraint was set from January 2000. All investigations which compared two or more types of polyethylene liners for THA were accessed. Only studies that clearly stated the nature of the liner were included. Data from 60 studies (37,352 THAs) were collected. 56% of patients were women. The mean age of patients was 60.0 ± 6.6 years, the mean BMI was 27.5 ± 2.0 kg/m^2^. The mean length of follow-up was 81.6 ± 44.4 months. Comparability was found at baseline between groups. XLPE and HXLPE liners in THA are associated with the lowest wear penetration (mm/year) and the lowest revision rate at approximately 7 years of follow-up.

## Introduction

Total hip arthroplasty (THA) is one of the most successful surgical procedures, with satisfactory clinical outcomes and survival rates of up to 90% over 15 to 20 years^1,2^. Despite the technical progress in THA, complications and failures still occur, including periprosthetic joint infection, instability, and aseptic loosening caused by polyethylene wear^3,4^.

Over the last decades, novel advances, such as the development of new implant designs and materials, have further decreased revision rates in THA^5^. In THA, different types of bearing surfaces can be divided into two major categories: hard-on-hard bearings (metal-on-metal, ceramic-on-metal, and ceramic-on-ceramic) and hard-on-soft bearings (metal-on-polyethylene, ceramic-on-polyethylene) are currently in use.

Metal-on-metal (MOM) surfaces have been employed in physically active young patients. However, MOM leads to a significant increase in metal ion concentrations in the body^6^. Furthermore, MOM may result in metal hypersensitivity and aseptic lymphocyte-dominated vasculitis^6^. Although no significant differences in functional and imaging outcomes were found for ceramic-on-metal bearings (COM) compared to MOM, chromium ion levels were significantly lower in the COM group after 3 years but increased after 5 years^6^. Cceramic-on-ceramic bearings provide good wear resistance, but this bearing carries an increased risk of liner and head breakage^7^. While ceramic-on-polyethene (COP) provides good mechanical properties and high resistance to scratch and deformation, it was criticised for its high cost and susceptibility to prosthetic head fracture^8^. Metal-on-polyethylene (MOP) are the most widely used THA bearings because of their relative safety and cost-effectiveness^8^. Low friction polymer materials such as polytetrafluoroethylene, poly 2-methacryloyloxyethyl phosphorylcholine, polycarbonate-urethanethe, polyether-ether-ketone, ultra-high molecular weight polyethylene (UHMWPE) and cross-linked polyethylene have outstanding mechanical properties and wear resistance^9^. Polyethylene is produced by the polymerization of ethylene, and a molecular weight of at least 1 million g/mole is defined as the standard^10^.

As UHMWPE used in THA liners may induce an imunological reaction to wear particles which can cause osteolysis and aseptic loosening, PE inlays have been cross-linked by thermal treatment (cross-linked polyethylene (XLPE), with improved wear resistance^5^.

Crosslinking is achieved by irradiating polyethylene at a dose higher than required for sterilisation, which is approximately 25 kGy^11^. Depending on the level of crosslinking and manufacturing process, several different cross-linked polyethylene types have been described. First-generation XLPE were irradiated with a dose between 50 and 100 KGy^12^. After initial promising results of XLPE, the cross-linking affected the mechanical properties of UHMWPE, compromising its toughness, ultimate mechanical properties, stiffness, and hardness. This was explained by the formation of free radicals during the manufacturing process, leading to oxidative changes in the XLPE with potentially decreased resistance to wear in the long term^8^. The free radicals in XLPE can be removed during the crosslinking process by annealing and remelting^10^. This procedure has led to moderately/modified cross-linked polyethylene (MXLPE) and highly cross-linked polyethylene (HXLPE), which mainly differ in the degree of irradiation (MXLPE: 50–75 kGy, HXLPE: 90 kGy), and thus the level of crosslinking. While remelted HXLPE (90 kGy) has good oxidation and wear resistance but poor fatigue properties, annealed HXLPE (90 kGy) shows good wear and fatigue resistance but has poor oxidation resistance^13^. Moderately cross-linked (50–75 kGy) and remelted UHMWPE (MXLPE) exhibits good oxidation resistance, with moderate wear and fatigue resistance^14^.

Kim et al. showed promising results of HXPLE liners in combination with delta ceramic heads, with annual wear rates of 0.022 mm/year^15^. However, despite zero revisions for wear-related problems and clinically nonsignificant wear rates, osteolysis is still observed in 35% of HXLPE THA in young patients at 16-year follow-up^16^.

Reduction of free radical production in liners is obtained by blending the liner with vitamin E (α-tocopherol). The infusion of Vitamin E chemically stabilizes the polyethylene by interrupting the oxidation cycle by decreasing the reactivity of reactive species^17^. However, the amount of Vitamin E that can be added is limited because Vitamin E in higher doses may interfere with the cross-linking process and thereby increase the wear rates^17^. Galea et al. showed significantly decreased wear of femoral heads (metal and ceramic) with vitamin E-diffused HXLPE compared to a moderately cross-linked and mechanically annealed UHMWPE in the first 5 years after THA^18^. However, the wear rates for both liners were very low (0.00–0.07 mm/year) and have not led to osteolysis or implant failures caused by aseptic loosening^18^. Comparable results were shown when polyethylene wear of MXLPE and HXLPE was compared with HXLPE-VEPE at 5 years^19^.

Given the large number of different types of liners, this network meta-analysis was conducted to compare the types of polyethylene liners and determine which is associated with the lowest wear penetration rates (mm/year) and with the lowest rate of revision.

The type of liners compared were the crosslinked ultra-high molecular weight polyethylene (CPE/UHMWPE), vitamin E infused highly cross-linked polyethylene (HXLPE-VEPE), modified cross-linked polyethylene (MXLPE), highly cross-linked polyethylene (HXLPE), cross-linked polyethylene (XLPE).

## Methods

### Eligibility criteria

All investigations that compared two or more types of polyethylene liners for THA published from January 2000 to July 2024 were accessed. Only studies that clearly stated the type of the liner were included. According to the Oxford Centre of Evidence-Based Medicine^20^, only clinical studies with levels I to III of evidence were considered. Articles in English and German language were eligible. Only studies that reported quantitative data under the endpoints of interest were considered.

### Search strategy

This study followed the PRISMA extension statement for reporting systematic reviews incorporating network meta-analyses of health care interventions: checklist and explanations^21^. The PICOD algorithm was established:

- P (Problem): End stage hip OA;
- I (Intervention): THA;
- C (Comparison): CPE/UHMWPE, HXLPE, HXLPE-VEPE, MXLPE, XLPE;
- (Outcomes): Rate of revision surgery, wear penetration (mm/year)
- D (Design): Comparative clinical investigations.

In July 2024, PubMed, Scopus, Embase, Google Scholar, and Cochrane databases were accessed. A time constraint was set from January 2000. Medical subject headings (MeSH) used for the database search are reported in the appendix.

### Selection and data collection

Three authors (FM, FH, LS) performed the database search. The resulting titles were screened by hand, and if suitable, the abstract and the full text were accessed. If the full text was not accessible or available, the article was not included. The bibliography of the included studies was also screened by hand to identify additional studies. A third senior author (NM) solved disagreements.

### Data items

Three authors (FM, FH, LS) performed data extraction. The following data at baseline were extracted: first author and year of publication and journal, length of the follow-up, number of patients with related mean age and BMI (kg/m^2^), number of women, side of surgery, and Harris hip score (HHS). The following data were collected at the last follow-up: inlay wear penetration per year (mm/year) and revision rate. Data were collected in Microsoft Office Excel version 16 (Microsoft Corp, Redmond, USA).

### Assessment of the risk of bias and quality of the recommendations

The risk of bias was evaluated in accordance with guidelines in the Cochrane Handbook for Systematic Reviews of Interventions^22^. Two reviewers (FM & LS) evaluated the risk of bias in the extracted studies. Randomised controlled trials (RCTs) were evaluated using the revised Risk of Bias assessment tool (RoB2)^22,23^ of the Cochrane tool for assessing the Risk of Bias in randomised trials (RoB)^24^. Non-RCTs were evaluated using the Risk of Bias in Nonrandomised Studies of Interventions (ROBINS-I) tool^25^. The figure of the ROBINS-I was elaborated using the Robvis Software (Risk-of-bias VISualization, Riskofbias.info, Bristol, UK)^26^.

### Synthesis methods

The main author (FM) performed the statistical analyses following the recommendations of the Cochrane Handbook for Systematic Reviews of Interventions^27^. Mean and standard deviation were used for descriptive statistics. For baseline comparability, the IBM SPSS software was used. The sum of squares and mean of squares were evaluated. Comparability was assessed through the analysis of variance (ANOVA), with P_ANOVA_ > 0.1 considered satisfactory. The network meta-analyses were made through the STATA/MP software (Stata Corporation, College Station, Texas, USA). Only studies which clearly stated the nature of the type of polyethylene of the liner were included in the analyses. The analyses were performed using the STATA routine for Bayesian hierarchical random-effects model analysis. Continuous variables were analysed using the inverse variance method, using the standardised mean difference (SMD) effect measure. Binary data were analysed through the Mantel–Haenszel method, with the Log Odd Ratio (LOR) effect measure. Edge, interval, and funnel plots were performed and analysed. The overall transitivity, consistency, heterogeneity, and the size of the treatment effect of interest within-study variance were evaluated. The overall inconsistency was evaluated through the equation for global linearity via the Wald test. In P_Wald_ values > 0.05, the null hypothesis could not be rejected, and the consistency assumption could be accepted at the overall level of each treatment. Confidence and percentile intervals (CI a d PrI, respectively) were each set at 95%. Edge, interval, and funnel plots were performed. Egger’s test assessed plot asymmetry, with values of P_Egger_ < 0.05 indicating statistically significant asymmetry. The Egger test is a linear regression of the intervention effect estimates on their standard errors weighted by their inverse variance.

## Results

### Study selection

**Fig. 1:**
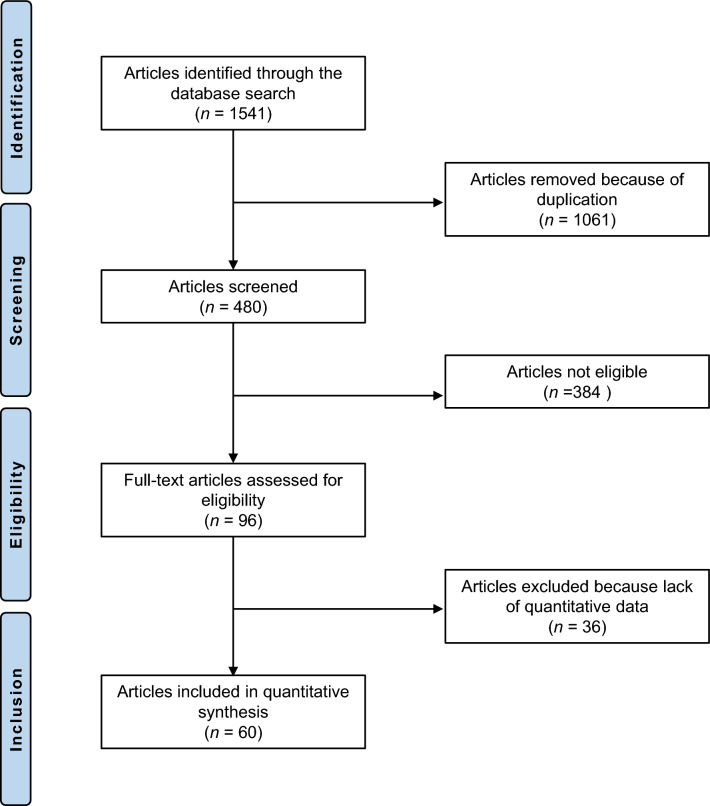
PRISMA flow chart of the literature search.

The systematic literature search resulted in 1541 articles. Of them, 1061 were identified as duplicates and therefore excluded. After reviewing the abstracts, a further 384 articles were discarded because they did not match the defined eligibility criteria: study design (*N* = 191), low level of evidence (*N* = 104), not comparing two or more types of polyethylene liners (*N* = 41), not clearly stated the nature of the liner (*N* = 31), and language limitations (*N* = 17). A further 36 studies were excluded as they missed quantitative data under the outcomes of interests. In conclusion, 60 comparative studies were included in the present investigation: 16 RCTs, 23 prospective, and 21 retrospective clinical trials. 52 (83.3%) of these investigations compared CPE/UHMWPE liner with HXLPE liner. The results of the literature research are shown in Fig. 1.

### Risk of bias assessment

**Fig. 2:**
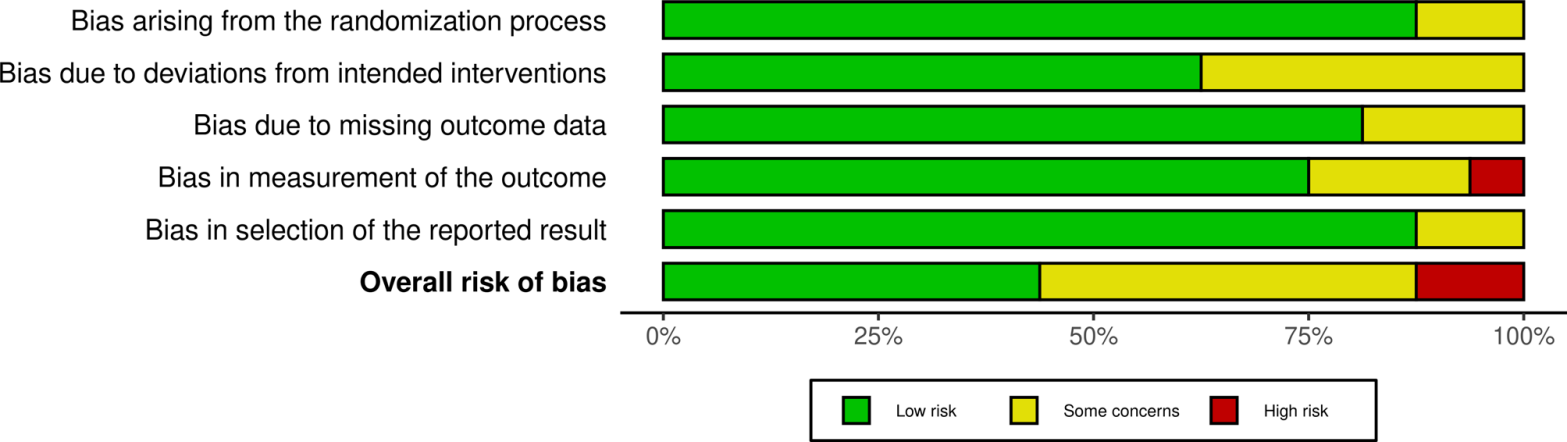
Cochrane risk of bias 2.0 tool (RoB2 tool).

To investigate the risk of bias for RCTs included in the present meta-analysis, the revised Risk of Bias assessment tool (RoB2) was performed. 27% (16 of 60) of the studies reviewed were RCTs. Most authors reported high-quality allocation concealments, resulting in comparable study groups at baseline, leading to an almost low risk of bias arising from the randomisation process. Some concerns about deviations from the intended intervention, missing outcome data, and selection of the reported outcome were detected in a few studies, leading to a low to moderate risk of bias. Given the lack of blinded assessors to intervention status, a high risk of bias was identified in the outcome measurement in two of the included investigations. In summary, the risk of bias graph indicates a low to moderate quality of methodological assessment of RCTs (Fig. 2).

**Fig. 3:**
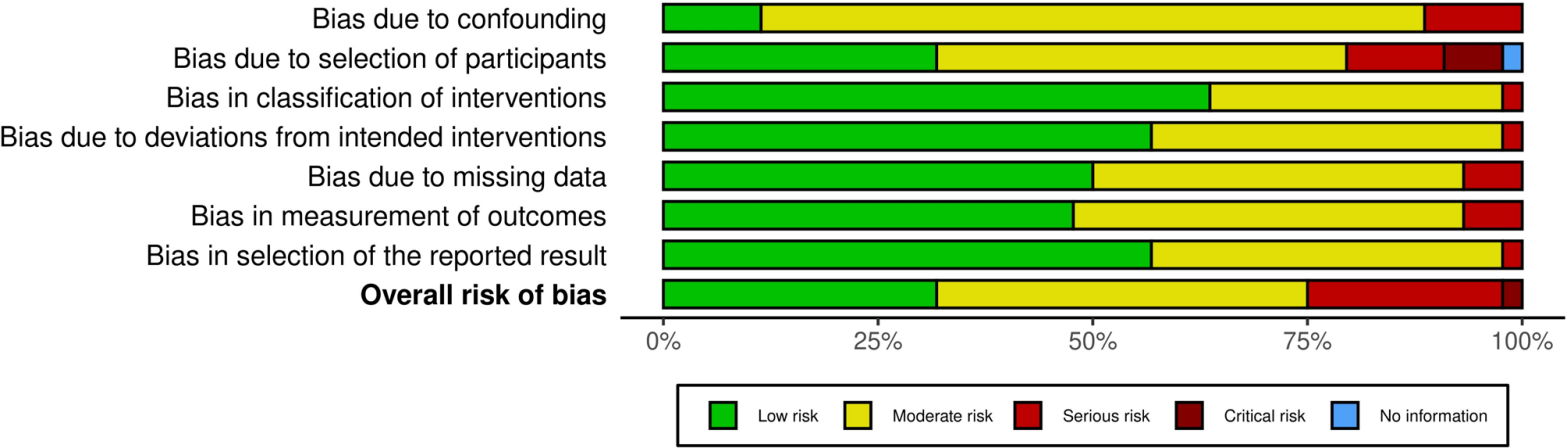
The ROBINS-I of non-RCTs.

The ROBINS-I was applied to investigate the risk of bias of non-RCTs. 73% (44 of 60) of the included investigation were NRSIs. 23% (10 of 44) studies were rated as having a serious risk of bias in at least one domain, but no critical risk of bias in any domain. One study was identified with a critical risk of bias in the domain of participant selection, but all other domains had a low to moderate risk of bias. Given the mainly good methodological quality of the included studies, the overall risk of bias was low to moderate (Fig. 3).

### Study characteristics

Data from 37,352 THAs were collected. 56% of patients were women. The mean patient age was 60.0 ± 6.6 years, the mean BMI was 27.5 ± 2.0 kg/m^2^. The mean length of follow-up was 81.6 ± 44.4 months. The generalities and demographic data of the included studies are shown in Table 1.

**Table 1:** Generalities and patient baseline data of the included studies.

Author, year	Journal	Design	Follow-up (*months*)	Type of polythylene	Patients (*n*)	Mean age	Women (%)
Atrey et al. 2017^28^	Bone Joint J	RCT	120	CPE/UHMWPE	34		
			120	HXLPE	29		
Beksaç et al. 2009^29^	Clin Orthop Relat Res	Retrospective	64	CPE/UHMWPE	41	53	43
			64	HXLPE	41	50	43
Bragdon et al. 2006^30^	J Arthroplasty	Prospective	45	CPE/UHMWPE	70	60	
			45	HXLPE	41	60	
			45	HXLPE	12	60	
Bragdon et al. 2013^31^	Clin Orthop Relat Res	Prospective		CPE/UHMWPE			
			84	HXLPE	174	60	49
Broomfield et al. 2017^32^	J Arthroplasty	Prospective	146	CPE/UHMWPE	27	68	45
			146	HXLPE	27	67	53
Bryan et al. 2019^33^	J Arthroplasty	Retrospective		CPE/UHMWPE	57	40	
				HXLPE	216	43	
Busch et al. 2020^34^	Arch Orthop Trauma Surg	RCT	60	HXLPE-VEPE	51	62	54
			60	HXLPE	43	62	56
Calvert et al. 2009^35^	J Arthroplasty	RCT		CPE/UHMWPE	59	61	59
				HXLPE	60	63	45
D’Antonio et al. 2005^36^	Clin Orthop Relat Res	Retrospective	64	CPE/UHMWPE	53	53	42
			59	HXLPE	56	57	49
Devane et al. 2017^37^	J Bone Joint Surg Am	RCT	132	CPE/UHMWPE	59	61	47
			132	HXLPE	57	61	37
Digas et al. 2003^38^	Clin Orthop Relat Res	RCT		CPE/UHMWPE	29	55	48
				HXLPE	32	54	53
Digas et al. 2004^39^	Clin Orthop Relat Res	RCT		CPE/UHMWPE	27	48	63
				CPE/UHMWPE	26	57	46
				HXLPE	27	48	63
				HXLPE	23	55	57
Digas et al. 2007^40^	Acta Orthop	Prospective		CPE/UHMWPE	32	48	66
				HXLPE	32	48	66
Dorr et al., 2005^41^	J Bone Joint Surg Am	Prospective	60	CPE/UHMWPE	37	65	54
			60	HXLPE	37	60	54
Engh et al. 2006^42^	J Arthroplasty	Prospective	68	CPE/UHMWPE	114	62	50
			68	HXLPE	116	63	56
Engh et al. 2012^43^	J Arthroplasty	RCT		CPE/UHMWPE	114	62	50
				HXLPE	116	63	56
Epinette et al. 2016^44^	J Arthroplasty	Retrospective	72	CPE/UHMWPE	8225		
			72	HXLPE	21,470		
Fredette et al. 2015^45^	Biomed Res Int	Retrospective	25	CPE/UHMWPE	35	53	37
			23	HXLPE	50	57	50
Fukui et al. 2013^46^	J Arthroplasty	Retrospective	127	CPE/UHMWPE	20	53	80
			125	HXLPE	36	57	94
Galea et al. 2019^47^	Bone Joint J	Prospective		HXLPE-VEPE	39	66	56
				MXLPE	34	63	59
Geerdink et al. 2006^48^	Acta Orthop	Prospective	56	CPE/UHMWPE	54	63	
			56	HXLPE	45	64	
Geerdink et al. 2009^49^	Clin Orthop Relat Res	RCT	96	CPE/UHMWPE	26	64	43
			96	HXLPE	22	64	35
Hanna et al. 2016^50^	Bone Joint J	Retrospective	158	CPE/UHMWPE	89	57	51
			157	HXLPE	88	56	90
Hopper et al. 2003		Retrospective	36	CPE/UHMWPE	50	60	
			34	CPE/UHMWPE	50	61	
			37	HXLPE	78	59	
			35	HXLPE	48	60	
Hopper et al. 2018^51^	Clin Orthop Relat Res	Prospective	176	CPE/UHMWPE	114	62	50
			188	XLPE	116	63	56
Ise et al. 2009^52^	J Arthroplasty	RCT	48	CPE/UHMWPE	26	60	96
			46	HXLPE	25	62	94
			45	HXLPE	23	63	100
Jassim et al. 2015^53^	Bone Joint J	Prospective	60	CPE/UHMWPE	124	63	56
			60	HXLPE	123	61	66
			60	HXLPE	121	63	56
Johanson et al. 2012^54^	Clin Orthop Relat Res	Prospective		CPE/UHMWPE	27	56	44
				HXLPE	25	55	52
Jonsson et al. 2015^55^	Bone Joint J	Prospective		CPE/UHMWPE	30	69	67
				CPE/UHMWPE	30	69	77
				HXLPE	30	70	67
				HXLPE	30	70	73
Karidakis et al. 2015^56^	Clin Orthop Relat Res	Retrospective		CPE/UHMWPE	45		
				XLPE	46		
				XLPE	48		
				XLPE	49		
Kawata et al. 2017^57^	J Orthop	Prospective		CPE/UHMWPE	26	60	
				HXLPE	25	62	
				HXLPE	23	63	
Keeney et al. 2015^58^	Hip Int	Retrospective		CPE/UHMWPE	84	40	43
				HXLPE	89	40	58
Kjaergaard et al. 2020^59^	Bone Joint J	RCT		HXLPE-VEPE	24	65	21
				HXLPE-VEPE	29	63	31
				XLPE	30	64	36
				XLPE	33	61	42
Krushell et al. 2005^60^	J Arthroplasty	Retrospective	50	CPE/UHMWPE	40	70	53
			48	HXLPE	40	69	53
Langlois et al. 2015^61^	Bone Joint J	Prospective		CPE/UHMWPE	50	66	55
				HXLPE	50	66	55
Leung et al. 2007^62^	J Arthroplasty	Retrospective	73	CPE/UHMWPE	40	62	58
			73	HXLPE	36	61	58
Mall et al. 2011^63^	Clin Orthop Relat Res	Retrospective	72	CPE/UHMWPE	50	43	
			99	HXLPE	48	47	
Manning et al. 2005^64^	J Arthroplasty	Prospective		CPE/UHMWPE	111	57	44
			44	HXLPE	70	61	50
Martell et al. 2003		RCT	28	CPE/UHMWPE	22	55	
			28	HXLPE	24	60	
Massier et al. 2020^65^	Acta Orthop	Prospective	72	CPE/UHMWPE	97	65	66
			72	HXLPE-VEPE	102	66	75
Miyanishi et al. 2008^66^	Arch Orthop Trauma Surg	Retrospective	50	CPE/UHMWPE	20	61	79
			28	HXLPE	95	67	83
Moon et al. 2020^67^	Hip Int	Retrospective	208	CPE/UHMWPE	22	50	45
			185	HXLPE	112	52	50
Morison et al. 2014^68^	J Arthroplasty	RCT	82	CPE/UHMWPE	21	51	48
			82	CPE/UHMWPE	21	52	36
			82	HXLPE	23	54	48
			82	HXLPE	22	51	55
Nakashima et al. 2013^69^	J Orthop Sci	Retrospective	157	CPE/UHMWPE	62	62	70
			138	HXLPE	69	62	82
Nikolaou et al. 2012^70^	J Bone Joint Surg Br	RCT	60	CPE/UHMWPE	36	53	50
			60	HXLPE	32	55	56
Oonishi et al. 2006^71^	J Arthroplasty	Prospective	28	CPE/UHMWPE	73	61	
			28	HXLPE	70	61	
Orradre Burusco et al. 2011^72^	Arch Orthop Trauma Surg	Prospective	70	CPE/UHMWPE	57	68	40
			65	HXLPE	50	65	36
Pang et al. 2015^73^	Clin Orthop Relat Res	Retrospective		CPE/UHMWPE	13	66	62
				HXLPE	13	61	62
Rajadhyaksha et al. 2009^74^	J Arthroplasty	Retrospective	75	CPE/UHMWPE	27	62	44
			71	HXLPE	27	60	32
Röhrl et al. 2005^75^	J Arthroplasty	Prospective	24	CPE/UHMWPE	20	67	75
			36	HXLPE	10	58	40
Röhrl et al. 2007^76^	Acta Orthop	Prosective	60	CPE/UHMWPE	20	70	40
			72	HXLPE	10	58	40
Sato et al. 2012^77^	J Orthop Res	Retrospective	145	CPE/UHMWPE	24	60	56
			145	CPE/UHMWPE	40	60	63
			73	HXLPE	72	62	85
			73	HXLPE	20	62	85
			73	HXLPE	275	62	85
Scemama et al. 2017^78^	Int Orthop	Prospective		CPE/UHMWPE	50	66	48
				HXLPE-VEPE	50	67	56
Sillesen et al. 2016^79^	Hip Int	Retrospective		HXLPE-VEPE	520	61	50
				MXLPE	457	62	50
Sköldenberg et al. 2019^80^	Bone Joint J	Prospective		CPE/UHMWPE	21	67	52
				HXLPE-VEPE	21	67	48
Teeter et al. 2017^81^	Can J Surg	RCT	156	CPE/UHMWPE	8	68	
			156	HXLPE	8	68	
Thoen et al. 2020^19^	Bone Joint J	RCT		HXLPE-VEPE	37	58	46
				MXLPE	31	61	48
Thomas et al. 2011^82^	J Bone Joint Surg Am	Prospective	84	CPE/UHMWPE	22	67	50
			84	HXLPE	22	68	55
Triclot et al. 2007^83^	J Bone Joint Surg Br	RCT	60	HXLPE	33	68	48
			60	XLPE	34	70	41
Tsukamoto et al. 2017^84^	J Arthroplasty	Retrospective	156	CPE/UHMWPE	38	58	89
			150	HXLPE	41	56	93

RCT, randomised controlled trial; CoCr, Cobalt-Chrome; CPE/UHMWPE, crosslinked ultra-high molecular weight polyethylene; HXLPE-VEPE, Vitamin E infused highly cross-linked polyethylene; MXLPE, modified cross-linked polyethylene; HXLPE, highly cross-linked polyethylene; XLPE, Cross-linked polyethylene.

### Baseline comparability

Between groups, baseline comparability in mean age, mean BMI, women:men ratio, side, length of the follow-up, and HHS was evidenced (Table 2).

**Table 2:** Baseline comparability.

Endpoint	Sum of square	Mean of square	P_ANOVA_
Mean age	260	65	0.2
Mean BMI (kg/m^2^)	3.1128	0.7782	0.9
Women (%)	0.19	0.05	0.1
Side Right (%)	0.00	0.00	0.9
Follow-up (months)	6482.417	2160.806	0.4
HHS	193.8582	48.4646	0.08

HHS, Harris hip score.

### Synthesis of results

**Fig. 4:**
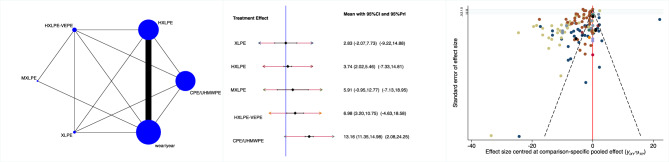
From left to right: edge, funnel, and interval plots of the comparison: wear penetration (mm/year).

XLPE, followed by HXLPE, demonstrated the lowest wear penetration (Fig. 4). The equation of global linearity found no statistically significant inconsistency in all comparisons (P_Wald_ = 0.2). The Egger test found no statistically significant asymmetry (P_Egger_ = 0.9).

**Fig. 5:**
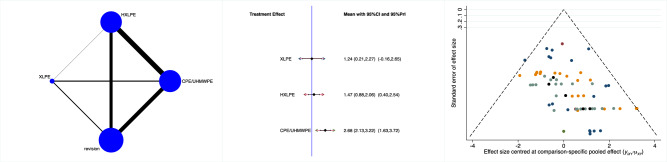
From left to right: edge, funnel, and interval plots of the comparison: revision.

XLPE, followed by HXLPE, demonstrated the lowest rate of revision at the last follow-up (Fig. 5). The equation of global linearity evidenced no statistically significant inconsistency in all comparisons (P_Wald_ = 0.9). The Egger test found no statistically significant asymmetry (P_Egger_ = 0.07).

## Discussion

The present study shows that XLPE followed by HXLPE demonstrated the lowest wear penetration and the lowest rate of revision after THA at a mean follow-up of 81.6 ± 44.4 months compared to other polyethylene liners (CPE/UHMWPE), HXLPE-VEPE, MXLPE).

Polyethylene is a complex material, and its morphological and mechanical properties are temporal and dependent on functional loading and environmental conditions^85^. UHMWPE is a linear (non-branching) semi-crystalline polymer, which can be described as a two-phase composite of crystalline and amorphous phases, which both influence the mechanical properties of the polymer^85^. While the crystalline phase provides modulus or stiffness to the material, the amorphous phase provides ductility and toughness^13^. Given its excellent wear resistance, high strength and biological inertness, UHMWPE remains the most commonly used bearing material in THA since its introduction in 1962^2,86,87^. However, particulate wear and the consequent osteolysis related to its wear debris and delamination wear from oxidation reduced the longevity of the UHMWPE implants, with subsequent aseptic loosening, with the need for revision surgery^13,86^. In light of these increasing demands in revisions, the necessity to develop longer-lasting, more resilient formulations of UHMWPE was obvious^85^. Osteolysis is an inflammatory process induced by exposure to wear particles of UHMWPE, which is in part consequent to the oxidation process and is even more evident when using gamma sterilisation in air. This has led to introducing gamma irradiation in an inert environment, using ethylene oxide and gas plasma^88^, significantly decreasing the wear rates in conventional UHMWPE.

Additionally, gamma radiation can break the C–C bonds of the polyethylene chain and induce cross-linking, which can potentially increase wear resistance^13,88^. Therefore, in the late 1990s, cross-linked—UHMWPEs (XPLE) were proposed for joint arthroplasties^13^. The initially used sterilisation dose of 25–40 kGy of gamma radiation was increased up to 100 kGy with a linear correlation to the degree cross-linking obtained^12,89^. With higher doses of radiation, the cross-link density did not increase further, and the mechanical properties of UHMWPE were affected, compromising toughness, ultimate mechanical properties, stiffness, and hardness, mainly caused by possible formation of free radicals during the manufacturing process, leading to oxidative changes in the XLPE^8^. For this reason, all first-generation XLPE were irradiated with a dose between 50 and 100 KGy^12^. Improvements in XLPE were achieved by increasing the irradiation dose and removing the free radicals trapped in the crystalline phase^89^. Two thermal treatments were used to remove free radicals in XLPE: remelting or annealing^89^. With remelting, it is possible to remove all free radicals. However, the process decreases its mechanical properties. Annealing does not alter the mechanical properties significantly, but it cannot remove all free radicals, and the oxidative process continues during storage and in vivo after implantation^13^.

In general, remelted HXLPE (90 kGy) has good oxidation and wear resistance but poor fatigue properties, while annealed HXLPE (90 kGy) shows good wear and fatigue resistance but has poor oxidation resistance^13^. Moderately cross-linked (50–75 kGy) and remelted UHMWPE (MXLPE) exhibits good oxidation resistance, with moderate wear and fatigue resistance^14^.

Supporting our results, several studies on the in vivo performance of acetabular components have shown significantly reduced wear rates for HXLPE compared with conventional polyethylenes^36,39,41,42,51,60,64,75,90^. However, the highly crosslinked annealed UHMWPE formulations may also undergo oxidation in vivo, as was shown in irradiation sterilised conventional UHMWPE^13^. Several studies reported that remelted HXLPE show little or no in vivo oxidation^91,92^, while annealed HXLPE do undergo oxidation in vivo^91–93^, with maximum oxidative degradation near the rims^91,92^. Complications are also reported in remelted HXLPE, such as rim fractures after short-term implantation (6 months to 3.8 years)^94–96^.

A second generation of HXLPE was developed to improve fracture resistance to maintain the wear resistance of the first-generation HXLPE while retaining the superior mechanical properties of conventional UHMWPEs^13^.

Several methods have been developed: mechanical deformation^97^, high-pressure crystallisation after melting HXLPE^98^, sequential annealing^99,100^, and incorporation of vitamin E^101–103^. Vitamin E-infused highly cross-linked polyethylene (HXLPE-VEPE) should reduce free radical production and stabilise polyethylene by interrupting the oxidation cycle, thereby decreasing the reactivity of radical species^17^.

Oral et al. showed promising early in vitro results compared with irradiated UHMWPE^101,102^. HXLPE-VEPE showed some oxidation on the surface, which stayed constant thereafter,

UHMWPE exhibited substantial oxidation in the subsurface region, which increased over time^102^. The hip simulator wear rate of HXLPE-VEPE showed a fourfold to tenfold decrease from that of conventional UHMWPE^101^. Studies comparing HXLPE-VEPE with UHMWPE or MXLPE showed similar results^78,79^. Six-year results in a recent RCT including 199 patients reported superior results of vitamin E blended HXLPE (0.028 mm/year) compared with UHMWPE (0.035 mm/year)^65^. Significantly decreased wear rates of vitamin E-diffused HXLPE compared to a moderately cross-linked and mechanically annealed UHMWPE coupled with metal and ceramic femoral heads were shown within the first 5 years after THA^18^. However, both liners showed very low rates of wear (0.00–0.07 mm/year), with osteolysis or implant failure from aseptic loosening^18^. Similar results were shown when polyethylene wear of MXLPE and HXLPE was compared with HXLPE-VEPE over 5 years^19^. A prospective, randomised, controlled, multicenter study also compared the mid-term results of HXLPE with HXLPE-VEPE, with no significant differences between the two cohorts regarding wear rate (HXLPE: 23.2 μm/year vs HXLPE-VEPE: 24.0 μm/year, *p* = 0.73) after 5-years follow up^34^. The antioxidative benefit of vitamin E is expected to become evident in long-term follow-up. However, the results of this present network meta-analysis indicate better performance of XLPE and HXLPE after 7 years of follow-up. Skoldenberg reported a significantly higher total migration and continuous proximal migration of the component in the VEPE group compared to conventional argon-gas gamma-sterilized PE, with a difference at 2 years of a mean of 0.21 mm^80^.

This network meta-analysis has several limitations. First, there is no consistency in the liner/head coupling, and different implants were used in the various studies. Metal-on-polyethylene (MOP) is the most widely used THA bearings. At the same time, ceramic-on-polyethene (COP) may provide better mechanical properties, but has higher costs and is susceptible to femoral head fractures^8^. There was also a high level of heterogeneity between the studies regarding surgical approaches, which is considered a source of bias. Most studies did not report data separately according to the femoral head size or did not report information on the size of the head. Therefore, it was not possible to conduct additional analyses based on head sizes. Several techniques exist to investigate wear penetration (e.g. radiostereometry, Martell method, Polyware^104,105^). However, given the between-studies heterogeneity in these techniques, the analyses were not conducted separately according to each method. The mean follow-up of the included studies was 81.6 ± 44.4 months, allowing only short to midterm conclusions about the wear rates and revision rates of each liner. Although aseptic loosening caused by polyethylene wear is frequent^3,4^, there is a lack of large prospective long-term clinical trials. In addition, different study types were analysed: 16 RCTs, 23 prospective, and 21 retrospective clinical trials. 83% (52 of 60) of these investigations compared CPE/UHMWPE liner with HXLPE liner. Second-generation HXLPE stabilised with vitamin E is underrepresented. Additional studies should be performed to overcome the current limitations, with long-term trials comparing more types of liner and including new-generation polyethylenes.

## Conclusion

XLPE and HXLPE liners in THA are associated with the lowest wear penetration (mm/year) and the lowest revision rate at approximately 7 years of follow-up.

## Supplementary Information

Supplementary Information.

## Data Availability

The datasets generated during and/or analysed during the current study are available throughout the manuscript.
