# Supplementary material for: Rate of revision and wear penetration in different polyethylene liner compositions in total hip arthroplasty: a Bayesian network meta-analysis
Source: Sci Rep. 2024 Sep 10;14:21162. doi: 10.1038/s41598-024-71326-1 (PMC11387631; doi:10.1038/s41598-024-71326-1)
Supplement: Supplementary file 1 — Supplementary Information. [file 41598_2024_71326_MOESM1_ESM.docx]

**Systematic literature search for:**

Rate of revision and wear penetration in different polyethylene liner compositions in total hip arthroplasty: a Bayesian network meta-analysis

**Concept 1: Total Hip Arthroplasty (THA)**

**Keywords:**

"Hip joint"

"End stage hip OA"

"Total Hip Arthroplasty"

"THA"

"Primary total hip arthroplasty"

**Mesh:**

"Hip Joint "[MESH]

"Osteoarthritis, Hip"[Mesh]

"Hip Joint/surgery"[Mesh]

"Surgical Procedures, Operative"[Mesh]

"Orthopedic Procedures"[Mesh]

"Arthroplasty"[Mesh]

"Arthroplasty, Replacement, Hip"[Mesh]

"Prosthesis Implantation"[Mesh]

"Hip Prosthesis"[Mesh]

**Concept 2: Liner types to compare**

**Keywords:**

"crosslinked ultra-high molecular weight polyethylene (CPE/ UHMWPE)"

"VitaminE-infused highly cross-linked polyethylene (HXLPE-VEPE)"

"modified cross-linked polyethylene (MXLPE)"

"highly cross-linked polyethylene (HXLPE)"

"Cross-linked polyethylene (XLPE)"

**Mesh:**

"Polyethylenes"[Mesh]

"ultra-high molecular weight polyethylene" [Supplementary Concept]

**Concept 3: Interest in Outcome**

**Keywords**

"Rate of revision surgery"

"wear penetration (mm/year)"

**Mesh:**

"Reoperation"[Mesh]

**Concept 1: Total Hip Arthroplasty (THA)**

"Hip Joint "[MESH] OR "Osteoarthritis, Hip"[Mesh] OR "Hip Joint/surgery"[Mesh] OR "Surgical Procedures, Operative"[Mesh] OR "Orthopedic Procedures"[Mesh] OR "Arthroplasty"[Mesh] OR "Arthroplasty, Replacement, Hip"[Mesh] OR "Prosthesis Implantation"[Mesh] OR "Hip Prosthesis"[Mesh] OR Hip joint OR End stage hip osteoarthritis OR Total Hip Arthroplasty OR THA OR Primary total hip arthroplasty

**AND**

**Concept 2: Liner types to compare**

"Polyethylenes"[Mesh] OR "ultra-high molecular weight polyethylene" [Supplementary Concept] OR crosslinked ultra-high molecular weight polyethylene OR Vitamin E infused highly cross-linked polyethylene OR modified cross-linked polyethylene OR highly cross-linked polyethylene OR Cross-linked polyethylene

**AND**

**Concept 3: Interest in Outcome**

"Reoperation"[Mesh] OR revision rate OR wear penetration

**Summary of the Search**

(("Hip Joint "[MESH] OR "Osteoarthritis, Hip"[Mesh] OR "Hip Joint/surgery"[Mesh] OR "Surgical Procedures, Operative"[Mesh] OR "Orthopedic Procedures"[Mesh] OR "Arthroplasty"[Mesh] OR "Arthroplasty, Replacement, Hip"[Mesh] OR "Prosthesis Implantation"[Mesh] OR "Hip Prosthesis"[Mesh] OR Hip joint OR End stage hip osteoarthritis OR Total Hip Arthroplasty OR THA OR Primary total hip arthroplasty) AND ("Polyethylenes"[Mesh] OR "ultra-high molecular weight polyethylene" [Supplementary Concept] OR crosslinked ultra-high molecular weight polyethylene OR Vitamin E infused highly cross-linked polyethylene OR modified cross-linked polyethylene OR highly cross-linked polyethylene OR Cross-linked polyethylene)) AND ("Reoperation"[Mesh] OR revision rate OR wear penetration)

**A time constraint was set from January 2000 to July 2023**
